# Home-Based Remedies to Prevent COVID-19-Associated Risk of Infection, Admission, Severe Disease, and Death: A Nested Case-Control Study

**DOI:** 10.1155/2022/4559897

**Published:** 2022-03-16

**Authors:** Benjamin Demah Nuertey, Joyce Addai, Priscilla Kyei-Bafour, Kingsley Appiah Bimpong, Victor Adongo, Laud Boateng, Kareem Mumuni, Kenneth Mibut Dam, Emilia Asuquo Udofia, Nana Ayegua Hagan Seneadza, Benedict NL Calys-Tagoe, Edem M. A. Tette, Alfred Edwin Yawson, Sari Soghoian, Gideon K. Helegbe, Rajesh Vedanthan

**Affiliations:** ^1^Tamale Teaching Hospital, Tamale, Ghana; ^2^Department of Community Health, University of Ghana Medical School, Accra, Ghana; ^3^Department of Medicine, Korle-Bu Teaching Hospital, Accra, Ghana; ^4^Department of Population Health, Department of Medicine, NYU Langone Health, New York, USA; ^5^Department of Obstetrics and Gynaecology, University of Ghana Medical School, Korle-Bu, Accra, Ghana; ^6^Department of Emergency Medicine, New York University School of Medicine, New York, NY, USA; ^7^Department of Biochemistry & Molecular Medicine, School of Medicine, University for Development Studies, Tamale, Ghana

## Abstract

**Objective:**

This study aimed at determining the various types of home-based remedies, mode of administration, prevalence of use, and their relevance in reducing the risk of infection, hospital admission, severe disease, and death.

**Methods:**

The study design is an open cohort of all participants who presented for testing for COVID-19 at the Infectious Disease Treatment Centre (Tamale) and were followed up for a period of six weeks. A nested case-control study was designed. Numerical data were analysed using STATA version 14, and qualitative data were thematically analysed.

**Results:**

A total of 882 participants made up of 358 (40.6%) cases and 524 (59.4%) unmatched controls took part in the study. The prevalence of usage of home-based remedies to prevent COVID-19 was 29.6% (*n* = 261). These include drinks (34.1% (*n* = 100)), changes in eating habits/food (33.8% (*n* = 99)), physical exercise (18.8% (*n* = 55)), steam inhalation (9.9% (*n* = 29)), herbal baths (2.7% (*n* = 8)), and gurgle (0.7 (*n* = 2)). Participants who practiced any form of home-based therapy were protected from SARS-CoV-2 infection (OR = 0.28 (0.20–0.39)), severe/critical COVID-19 (OR = 0.15 (0.05–0.48)), hospital admission (OR = 0.15 (0.06–0.38)), and death (OR = 0.31 (0.07–1.38)). Analysis of the various subgroups of the home-based therapies, however, demonstrated that not all the home-based remedies were effective. Steam inhalation and herbal baths were associated with 26.6 (95% CI = 6.10–116.24) and 2.7 (95% CI = 0.49–14.78) times increased risk of infection, respectively. However, change in diet (AOR = 0.01 (0.00–0.13)) and physical exercise (AOR = 0.02 (0.00–0.26)) remained significantly associated with a reduced risk of infection. We described results of thematic content analysis regarding the common ingredients in the drinks, diets, and other home-based methods administered.

**Conclusion:**

Almost a third of persons presenting for COVID-19 test were involved in some form of home-based remedy to prevent COVID-19. Steam inhalation and herbal baths increased risk of COVID-19 infection, while physical exercise and dietary changes were protective against COVID-19 infection and hospital admission. Future protocols might consider inclusion of physical activity and dietary changes based on demonstrated health gains.

## 1. Introduction

The novel coronavirus which causes an atypical respiratory tract infection was discovered in December 2019 in the Chinese city of Wuhan [1]. Its spread was so rapid that, as at March 11, 2020, about 111 countries worldwide were affected, with more than one million cases leading to the World Health Organization (WHO) declaring it a pandemic [2]. Ghana recorded her index cases on 12^th^ of March 2020, and the country had recorded more than 127,000 cases as at end of September 2021 with more than 1150 mortalities [3, 4].

There have been varying presentations of this disease. In a systematic review, Alene et al. [5] reported that a quarter of cases have been asymptomatic in nature. Among symptomatic cases, most reported cases have had mild disease which did not require hospitalization [5]. Fever, dry cough, headache, and fatigue have been some common presentations among symptomatic cases [6].

A number of strategies have been postulated to help in the prevention of COVID-19 and also decrease risk of infection, hospitalization, and death. Before the advent of vaccines for this disease, the world had to rely solely on measures such as quarantine, isolation, and other infection control measures to prevent the spread of the disease. Infection control measures that have been documented to decrease the spread of the disease include the use of appropriate face masks, eye protection, and physical distancing that is one meter or greater [7].

Home-based strategies such as intake of certain foods and food supplements have also been suggested to have possible protective and therapeutic effects against COVID-19 [8, 9]. A recent study also documented the use of home-based medicinal plants in the prevention of COVID-19 as well as the treatment of its associated respiratory symptoms [10]. In Africa, a study in Morocco showed that more than half of the study subjects used home-based medicinal plants during this current pandemic to boost their immune system and treat respiratory tract infections that are associated with the COVID-19 infection [11]. However, some of these complementary measures for preventing the infection have been associated with side effects such as nausea, vomiting, and diarrhoea [12].

In our setting, not much has been documented about the use of home-based remedies in the prevention of COVID-19. The usefulness or otherwise of these methods in reducing the risk of infection, hospital admission, severe disease, and death is yet to be studied in Northern Ghana. This study thus aimed at ascertaining the various types of home-based remedies employed in the prevention of COVID-19 and its relevance in reducing the risk of infection, hospital admission, and severe disease.

## 2. Methods

### 2.1. Study Design

The study design is an ongoing open cohort of all participants who presented from June 2020 to December 2020 to be tested for COVID-19 (using PCR) at the Infectious Disease Treatment Centre (Tamale) and were followed up for six weeks or to time of death, depending on whichever came first. However, all those who tested positive had an extended follow-up period for a maximum of 12 weeks or till complete resolution of symptoms, whichever came first. A nested case-control study was designed from this cohort of participants. All participants who tested positive and negative were eligible to be selected as cases and controls. Participants were interviewed at time of testing and when the result of PCR test became available. Upon discharge from the COVID-19 pathway of care, phone calls were made to the participants to collect further information over the period of the study. Clinical data of management within the COVID-19 pathway of care were included in the study. Questions were asked about specific exposures two weeks prior to testing for SARS-CoV-2.

### 2.2. Study Site

The study was carried out in a tertiary facility setting in the Northern Region of Ghana. The Infectious Disease Treatment Centre (IDTC) for the Northern Region is located within the premises of the Tamale Teaching Hospital. The centre receives and manages COVID-19 patients referred from all facilities in the northern sector of the country. Walk-in patients are also accepted. Recruitments were open, and participants were allowed to join within the study period. The last day of recruitment was 7^th^ December 2020, and the last day of follow-up was in February 2021. Initial assessment was carried out at the IDTC or the Infectious Disease Isolation Centre which also doubled as the Infectious Disease Clinic during the first wave of the pandemic in Ghana. PCR testing was carried out in two reference laboratories: the Zonal Public Health Reference Laboratory (ZPHRL) and the Central Veterinary Service Laboratory (CVSL) which currently have level 2 and 3 biosafety facilities, respectively.

### 2.3. Participants

All participants presenting for SARS-CoV-2 PCR testing, symptomatic or asymptomatic at time of presentation, were eligible for inclusion in the cohort. There were no age or sex restrictions in the original cohort. For children less than 18 years, parents or guardians provided answers to the questions and follow-up calls were made to such guardians. Case ascertainment was strictly by SARS-CoV-2 PCR results. The goal was a 1 : 2 case-to-control ratio; however, at the peak of the first wave, we had in some weeks more cases than controls making it difficult to achieve the case-to-control ratio. This period of recruitment coincided with the first wave of the pandemic in the Northern Region. A total of 882 participants made up of 358 (40.6%) cases and 524 (59.4%) unmatched controls were included in the study.

### 2.4. Study Variables

We asked if participants were engaged in any home-based therapy or remedy aimed at preventing COVID-19 within the past two weeks prior to testing for SARS-CoV-2 infection. Among participants who engaged in such practices, further enquiries were made to determine the mode of application/administration and the active ingredients in the preparation. This aspect of the study was approached qualitatively by using open-ended questions to identify all the possible home-based remedies practiced by participants. We also determined the pattern of administration of the various ingredients. Another variable of interest was the severity of infection. Severe COVID-19 was as defined by the WHO [13]. Severe COVID-19 was defined by the presence of any of these criteria: “oxygen saturation <90% on room air” or “respiratory rate >30 breaths/min in adults and children >5 years old; ≥60 breaths/min in children <2 months old; ≥50 in children 2–11 months old; and ≥40 in children 1–5 years old,” or “signs of severe respiratory distress (accessory muscle use, inability to complete full sentences, and, in children, very severe chest wall indrawing, grunting, central cyanosis, or presence of any other general danger signs).” Critical disease was defined by the following criteria: “acute respiratory distress syndrome (ARDS), sepsis, septic shock, or other conditions that would normally require the provision of life-sustaining therapies such as mechanical ventilation (invasive or noninvasive) or vasopressor therapy.”

Forms of disease presentation, other than severe and critical, were classified as nonsevere (asymptomatic/mild/moderate). Place of management was classified as inpatient management and outpatient management. Based on clinical criteria, patients were either admitted or managed at home. This decision was purely clinical and carried out by the team of healthcare practitioners on duty. Regular intake/administration was defined as daily intake/administration and irregular as any other pattern of intake/administration other than daily intake.

Further questions were asked about potential confounders such as face mask usage, age, healthcare worker status, vitamin C, and other immune booster use and adjustments made for potential confounding in the analysis. All measurements and assessments, including PCR tests, were carried out using standard procedures. A test was declared positive if PCR detected both SARS-CoV-2 and pan-CoV. The test was repeated if only SARS-CoV-2 was detected while pan-CoV was not detected. It was declared negative for SARS-CoV-2 if SARS-CoV-2 was not detected by RT-PCR.

### 2.5. Ethical Considerations

Ethical clearance for this study was obtained from the Tamale Teaching Hospital Ethical Review Committee (reference ID: TTHERC/30/09/20/07), and permission was obtained from the management of Infectious Disease Treatment Centre (Tamale) to carry out the study. The study followed the principles of the Declaration of Helsinki. Informed consent was verbally obtained from all participants.

### 2.6. Analysis

Data generated were cleaned and exported from the MS Excel data management template to STATA version 14 by StataCorp, California, USA, for analysis. For the quantitative variables, background characteristics were displayed using cross tabulations and chi-squared *p* values were obtained. Logistic regression analysis was carried out to determine the risk of testing positive by PCR among participants who engaged in a home-based remedy. Multivariate logistic regression analysis was carried out adjusting for potential confounders such as age, mask use, intake of immune booster, and healthcare worker status. The analyses were repeated for risk of severe or critical disease, risk of hospital admission, and risk of death from COVID-19. Subgroup analysis was carried out for those who engaged in home-based remedies. This was done for the individual methods of administration of the home-based methods. This was to determine the risk of infection among those who practiced steam inhalation, herbal baths, drinks, diet changes, and physical exercise to prevent COVID-19 infection. In subgroup analyses where study was not sufficiently powered to determine significant findings, these analyses were excluded. The qualitative data were thematically analysed using manual coding with themes being mode of application and subthemes being the patterns of use and the active ingredients. Qualitative data were displayed in tables.

## 3. Results

### 3.1. Background Characteristics of Study Participants

Table 1 displays the background characteristics of study participants. A total of 882 participants, comprising 358 (40.6%) cases and 524 (59.4%) controls, took part in the study. The nonresponse rate within the study period was 2.8% (*n* = 25) of which 23 tested negative for SARS-CoV-2. Main reasons for nonresponse included refusal to participate, travelling outside making it impossible for initial assessment, and phone numbers unreachable. The median age was 32 (IQR = 28–38) years. A total of 347 (39.3%) participants were nonhealthcare workers, and 261 (29.6%) of all participants administered some home-based remedies to prevent COVID-19 within two weeks prior to testing for COVID-19. Regarding the use of home-based methods against COVID-19, 55 (21.1%) of those who admitted to the use of home-based methods and 303 (48.8%) of those who did not use home-based methods tested positive for SARS-CoV-2.

### 3.2. Method of Application of Home-Based Remedies against COVID-19

Participants used home-based remedies in various forms. The main methods of application of home-based remedies were mainly as drinks (34.1% (*n* = 100)), by eating (food) (33.8% (*n* = 99)), physical exercise (18.8% (*n* = 55)), steam inhalation (9.9% (*n* = 29)), herbal baths (2.7% (*n* = 8)), and gurgle (0.7 (*n* = 2)).  Table 2 displays the differential proportion of methods used for home-based remedies segregated by cases and controls.

### 3.3. Methods of Application, Patterns of Use, and Ingredients Used in Home Remedies to Prevent COVID-19

A thematic content analysis was conducted to explore the methods of application, patterns of use, and main ingredients in the home-based remedies administered in the two weeks prior to testing for COVID-19. The results are described in Table 3. Steam inhalation takes the form of pure steam, menthol-based steam, or various herbal concoctions. Others practiced the use of certain specified herbal and other unspecified substances in bathing. Neem tree leaves were the main ingredient in inhalation therapy and baths. These warm baths and steam inhalation were based on the general belief that heat kills SARS-CoV-2.

Various drinks and herbal concoctions were imbibed to boost the natural immunity to fight any strain of SARS-CoV-2 that might have invaded the human body. Ginger is “hot” and is believed to burn the virus as well as contain properties that prevent the virus from causing harm to the body. Hence, ginger has been the dominant ingredient in most of the drinks. Ginger with or without sweeteners or ginger in combination with other fruits, herbs, or seeds was commonly practiced as a preventive drink against COVID-19. Citrus fruits with sour properties such as lemon and lime were also believed to prevent COVID-19 and as such were active ingredients in many drinks taken to prevent COVID-19. Ginger and lemon were used in various combinations as was ginger and garlic with or without sweeteners. Beverages made from *Hibiscus* leaves locally called “*sobolo*” became popular for fighting COVID-19.

Other drinks taken to prevent COVID-19 included boiled neem tree leaves, guava leaves, pineapple peels, and moringa leaves independently or in combinations with each other. Spices such as Aidan fruit locally called “*prekese*” was taken as drink or mixed with other spices. Cloves were also common in some of the drinks. Others also adopted a lifestyle of drinking some types of teas with the aim of preventing COVID-19. The temperature of water used for drinks was important. Warm temperature of the drinks is preferred due to the widely held belief that SARS-CoV-2 survives best in cold environment and is destroyed by heat.

Dietary changes such as adopting the habit of eating fruits and vegetables at a level not previously done, with the aim of preventing COVID-19, were common. Warm saline gurgle and chlorhexidine solution gurgle were also identified in the qualitative analysis as other approaches to prevent COVID-19.

### 3.4. Effectiveness of Home-Based Remedies against COVID-19

Logistic regression showed that those who practiced any form of home-based remedies were significantly protected from SARS-CoV-2 infection (OR = 0.28 (0.20–0.39)), risk of severe or critical COVID-19 (OR = 0.15 (0.05–0.48)), hospital admission (OR = 0.15 (0.06–0.38)), and death (OR = 0.31 (0.07–1.38)). After adjusting for known confounders such as age, face mask use, intake of immune booster, and healthcare worker status, administration of any home-based remedy prevented infection and hospital admission, but not severe disease and death (Table 4).

Subgroup analyses of the effectiveness of the method of administration of the home-based remedies showed that not all the home-based remedies were effective. For example, steam inhalation and herbal baths were associated with increased risk of infection, 26.6 (95% CI = 6.10–116.24) and 2.7 (95% CI = 0.49–14.78) times, respectively, compared to those who did not use any home-based remedies (Table 5). Change in diet or food eaten and exercise remained significantly associated with a reduced risk of infection after adjusting for known confounders such as age, face mask use, immune booster use, and healthcare worker status. Conversely, the use of these types of home-based remedies did not appear to be protective against hospital admission (Table 5).

## 4. Discussion

This study examined the use of home-based remedies in the prevention and adjunctive treatment of COVID-19. We established that nearly thirty percent of the participants used some form of home-based remedies. Among those who reported the use of home-based remedies, at least one in every five was diagnosed with SARS-CoV-2 compared to approximately one in every two of those who did not report the use of home-based remedies. Home-based remedies reported were protective against infection and admission, but not severe disease and death. Some home-based remedies such as change in diet or foods eaten and physical exercise were significantly associated with a reduced risk of infection, while measures such as steam inhalation and herbal baths were associated with increase in infection.

The use of home-based remedies in the prevention and management of COVID-19 has been reported by other authors in other jurisdictions [10, 11]. In our study, we noted that 29.6% of participants used some form of home-based remedies. This was lower than the 80% reported by Villena-Tejada et al. [10] in Peru, 64% reported by Al Najran et al. [14] in Saudi Arabia, and 67% reported in Morocco [11]. This difference could possibly be explained by the composition of the study subjects as about 60% of our participants were healthcare workers.

The route of administration of the various home-based remedies in our study was not different from what was reported by earlier authors. Steam inhalation, baths, and oral routes were the routes of administration used for the various home-based remedies in our study. Similar routes were reported by Chali et al. in Ethiopia [15]. Neem leaves were one of the commonly used home-based remedies in our study. It was boiled and taken warm, and some participants bathed with it. Neem and its active ingredients have been shown to be potent in the prevention of diseases through the antioxidant and anti-inflammatory properties [16]. Bioactive components of the neem leaves such as oleanolic acid, ursolic acid, and methyl eugenol have been shown to effectively inhibit the binding of spike glycoprotein of SARS-CoV-2, thus preventing viral attachment [17]. It is thus not surprising that neem tree leaves were commonly used in our study as a home-based remedy against COVID-19.

Ginger was another home-based remedy commonly used in our study. Previous authors have reported that ginger has been used in various places as preventive measure against COVID-19 infection [10, 14]. In our study, some participants took it as a drink with or without sugar or honey, whereas others took it as part of other remedies such as garlic, lemon, and hibiscus. It is known that ginger contains biologically active compounds such as zingerone, gingerenone A, and geraniol, which have antiviral activities against SARS-CoV-2 and influenza virus [18]. Also, there are clinical trials underway to study the effect of ginger on clinical presentations in patients with COVID-19 infection [19]. The anti-inflammatory properties of *Hibiscus* have been demonstrated in literature [20]. In our study, this was a component of some of the drinks commonly used, together with ginger with or without sugar or honey or extra fruits such as lemon, lime, or pineapple. Bioactive compounds such as quercetin and kaempferol in *Hibiscus* have also been shown to have anti-inflammatory and antioxidant properties [21, 22].

Moringa leaves were taken in various forms, as a drink with plain moringa leaves or moringa leaves with cocoa powder and cinnamon, with or without honey. Moringa has also been seen in the literature to have antiviral, antidiabetic, anticancer, and anti-inflammatory properties [23–25]. Bioactive components of the moringa leaves, such as apigenin and ellagic acid, have also been documented to have binding ability to nonstructural proteins (nsp) 9 and 10 of SARS-CoV-2 virus. These proteins have been implicated in the abnormal neutrophil activity and cytokine response seen in the pathogenesis of the COVID-19 infection; thus, the binding of these bioactive compounds to the proteins provides an antiviral property for the bioactive chemicals in moringa leaves [26].

Physical exercise has also been shown to decrease the occurrences of acute respiratory infections [27]. Exercise was significantly associated with a decreased risk (AOR = 0.02 (0.00–0.26)) of COVID-19 infection in our study. This is well supported by the literature as exercise has been seen to boost the immune system, thus reducing the risk of COVID-19 infection [28, 29]. Mechanisms which have been postulated to support this include enhanced immune competence and immunovigilance [27]. The practice of a change in diet or foods eaten was also significantly associated with decreased risk of COVID-19 infection in our study (AOR = 0.01 (0.00–0.13)). This practice involved intake of more fruits and vegetables than routinely done by participants who practiced this form of home remedy. Other dietary changes included the addition of certain spices and seeds in diet such as black seed oil, ginger, and Aiden fruit (prekese). Earlier authors have revealed that the intake of fruits and vegetables is vital in the prevention of this infection as they have been shown to boost the immune system [30, 31]. The use of steam inhalation as a home-based remedy has not been established to help in the prevention and treatment of COVID-19 infection [32].

## 5. Study Limitations

Generally, factors such as healthcare worker status, intake of other commercially prepared immune boosters and vitamin C, and the use of face mask were determined a priori to be confounders in the relationship between home-based methods and potential effects on SARS-CoV-2 infection, severe disease, admission, and death. Steps were taken to include these in the data collection and adjusted for. This is a limitation that future studies should aim at avoiding. Also, the study was not sufficiently powered due to low numbers of participants engaged in some methods such as gurgle, baths, and steam inhalation to allow for subgroup analysis of the effect of some of these methods on the risk of death, admission, and severe disease. Also, the study was not sufficiently powered to determine the effect of the ingredients used in the preparation of the home-based methods. Subgroup analysis of these methods and ingredients might have demonstrated effects on risk of infection, severe disease, admission, and death. Studies with larger sample sizes could include these variables and test for effects to inform future practice.

## 6. Conclusion

Nearly a third of persons presenting for the COVID-19 test used some form of home remedies to prevent COVID-19. On the whole, persons who practiced home-based therapy were protected from infection, severe disease, admission, and death from COVID-19. However, not all the methods of home-based therapy were effective. Our evidence supports physical exercise, deliberate inclusion of fruits and vegetables in diets, and drinking of fruit juices or home-based juices as effective methods for the prevention of SARS-CoV-2 infection. The study provides empirical evidence to discourage the use of steam inhalation in its various forms and herbal baths as means of preventing COVID-19; these methods increase your chances of getting COVID-19. The present study provides empirical and contextual evidence for the use of specific interventions to inform adjunctive therapy for COVID-19. Larger sample sizes will be required to conduct subgroup analyses on specific home-based remedies to determine inclusion or exclusion as adjunctive therapy.

## Figures and Tables

**Table 1:** Background characteristics of study participants segregated by type.

	Administration of home-based remedies within the last two weeks			
	*N*	No home-based remedy	Administered home-based remedy	Chi (*p* value)
*n* (%)	*n* (%)
*All participants*	882	621 (70.4)	261 (29.6)	
*SARS-CoV-2 test by PCR*				
Tested negative	524	318 (51.2)	206 (78.9)	58.55 (<0.001)
Tested positive	358	303 (48.8)	55 (21.1)	

*Sex*				
Female	371	267 (43.2)	104 (39.9)	0.8479 (0.357)
Male	508	351 (56.8)	157 (60.2)	

*Age (years)*				
Less than 20	48	40 (6.5)	8 (3.1)	13.15 (0.022)
20–29	292	203 (32.8)	89 (34.1)	
30–39	353	236 (38.1)	117 (44.8)	
40–49	95	68 (11.0)	27 (10.3)	
50–59	53	37 (6.0)	16 96.1)	
60 and above	39	35 (5.7)	4 (1.5)	

*Healthcare worker status*				
Healthcare professional	535	345 (55.6)	190 (72.8)	22.89 (<0.001)
Not a healthcare worker	347	276 (44.4)	71 (27.2)	

Severity of disease				
Asymptomatic, mild, moderate	834	576 (92.8)	258 (98.9)	13.27 (0.882)
Severe-critical	48	45 (7.3)	3 (1.2)	

*Management option*				
Outpatient (homecare)	806	550 (88.6)	256 (98.1)	21.14 (<0.001)
Inpatient	76	71 (11.4)	5 (1.9)	

*Outcome of care*				
Alive	865	606 (97.6)	259 (99.2)	2.64 (0.104)
Died	17	15 (2.4)	2 (0.8)	

**Table 2:** Method of administration of home-based remedies to prevent COVID-19.

Mode of administration (multiple response analysis)	All	Controls	Cases	Chi (*p* value)
	*N*	*n* (%)	*n* (%)	
*Method of home-based remedy administration*				
Steam inhalation	29	2 (6.9)	27 (93.1)	145.62 (≤0.01)
Herbal baths	8	3 (37.5)	5 (62.5)	
Drink	100	72 (72.0)	28 (28.0)	
Eating/in diet	99	98 (99.0)	1 (1.0)	
Exercise	55	54 (98.2)	1 (1.8)	
Gurgle	2	0 (0.0)	2 (100.0)	

**Table 3:** Qualitative analysis of modes, pattern, methods, and ingredients of home remedies' practices as prevention of COVID-19.

Home-based remedies administered within the past two weeks prior to testing for COVID-19	
Mode of administration	Method of administration and main ingredients

Steam inhalation	(i) *Pure steam inhalation*: this is the most dominant theme with almost half of all steam inhalations in our study were practiced using pure steam. (ii) *Various herbal concoctions used in steam inhalation*: this is the next dominant theme of method of practice of steam inhalation. There are subthemes under this approach which are described below: (a) *Neem tree leaves alone* was the most common method under this subtheme (b) *Various combinations including neem tree leaves*, for example: (1) Neem tree and lime (2) Neem tree and lemon (3) Neem tree and pawpaw leaves (4) Neem tree leaves plus ginger and pineapple (5) Neem tree leaves plus pineapple peels, cloves, and lemon (c) *Unspecified herbs* used in steam inhalation (iii) *Menthol-based steam inhalation* using menthol crystals or menthol-based preparations.

Baths	(i) *Herbal baths*: this is also the most dominant theme with almost half of all baths practiced using this method. The herbs used for herbal baths included the following: (a) *Neem tree leaves alone* was the most common method under this subtheme (b) *Neem tree and pawpaw leaves* (ii) *Unspecified herbs*

Drinks	*Ginger-based drinks*: ginger due to its burning properties is believed to burn SARS-CoV-2 and is a commonly practiced method of most of the home remedies. It was found to be taken in the following ways: (i) *Ginger drink* with or without sugar or honey (ii) *Ginger as part of other remedies* such as garlic, lemon, and hibiscus *Lemon-based drinks*: this a dominant team with more than a quarter of all home-based remedies having lemon in the mixture. The following subthemes of lemon used were found: (i) Lemon in warm water with or without sugar or honey (ii) Undiluted lemon juice (iii) Lemon and ginger mixture with or without sugar or honey (iv) Lemon together with other fruits (v) Lemon, ginger, garlic, and honey *Lime-based drinks:* this is one of the dominant themes. Lime taken to prevent COVID-19 is usually in the following forms: (i) Lime in warm water with or without sugar or honey (ii) Lime and ginger in warm water with or without honey (iii) Lime juice in undiluted form *Locally made herbal boiled drinks*: (i) *Neem tree leaves drink:* neem tree leaves boiled and taken warm (ii) *Guava leaves:* guava leaves drink taken alone or with other herbs (iii) *Pineapple peels boiled* (v) *Moringa* leaves: this is taken in various forms: (a) Plain moringa leaves drink (b) Moringa leaves with cocoa powder and cinnamon with or without honey *Spices mixed drinks* (i) Aidan fruit, locally called “prekese,” taken as drink or mixed with other spices (ii) Cloves taken as drink or mixed with ginger and other home-based remedies *Fruit juices*: in various forms and combinations of fruits; pineapple, oranges, and mixed fruit drinks. *Hibiscus-based drinks*: dried *Hibiscus* leaves and ginger drink, locally called “sobolo,” is widely believed to prevent and/or cure COVID-19. The *Hibiscus*-based drinks were taken in the following forms: (a) *Hibiscus* and ginger with or without sugar or honey (b) *Hibiscus* and ginger with fruits such as pineapple and/or lemon or lime (c) *Hibiscus* and ginger with spices such as Aidan fruit, locally called “prekese” *Teas*: increased tea intake or newly adopted lifestyle of tea intake to prevent COVID-19 was also practised by some. In all instances, the teas are taken warm. The commonest types of tea taken included the following: (a) Locally made herbal tea (b) Lemon and ginger tea (c) Other plain tea *Warm water*: warm water is taken by many because of the widely held belief that SARS-CoV-2 survives best in cold environment and is destroyed by heat. The methods of taking the water are either one of the following ways or both: (a) Warm water (b) Cucumber in water *Apple cider vinegar:* this is taken in warm water or mixed with other home-based remedies such as honey and lemon.
Food eaten or changes in diet	*Dietary changes*: changes in the content of the diet was a common theme in more than 30% of all who reported some home-based remedies: (i) Changes in the content and quantity of diet (ii) Adopting a healthy eating habit *Fruit intake*: more than half of all who reported a home remedy cited a change in fruit intake with the following pattern or subthemes: (i) Started taking fruits at least once each day (ii) Increased the number and types of fruits consumed daily *Vegetables:* some participants report a change in the consumption pattern of vegetables: (i) Increase in vegetable intake (ii) Increase in fruit and vegetable intake *Ginger chewed* with or without sugar in raw or dried forms. *Other dietary changes*: this includes addition of certain spices and seeds in diet such as black seed oil, ginger, and Aiden fruit (prekese).

Exercise	Exercise of all forms as the main change of habit to prevent COVID-19.

Gurgle	Gurgle was the least dominant theme in the following forms: (i) Warm saline water gurgle (ii) Chlorhexidine solution in warm water gurgle

^*∗*^One person stated resting well.

**Table 4:** Logistic regression showing the odds of infection, severe disease, admission, and death associated with the administration of any home-based remedy to prevent COVID-19.

Characteristics	Univariate logistic regression	Multivariate logistic regression	AOR [95% CI]	*p* value
	OR [95% CI]	*p* value		
Risk of PCR-positive SARS-CoV-2 test	0.28 [0.20–0.39]	<0.001	0.38 [0.26–0.57]	<0.001
Risk of severe or critical COVID-19	0.15 [0.05–0.48]	0.002	0.40 [0.11–1.48]	0.169
Risk of admission	0.15 [0.06–0.38]	<0.001	0.29 [0.11–0.74]	0.009
Risk of death from COVID-19	0.31 [0.07–1.38]	0.124	2.82 [0.60–13.3]	0.189

*Sex*				
Female	—			
Male	1.14 [0.86–1.54]	0.358		

*Age (years)*				
Less than 20	—			
20–29	2.19 [0.99–4.88]	0.054		
30–39	2.37 [1.12–5.47]	0.025		
40–49	1.99 [0.82–4.79]	0.127		
50–59	2.16 [0.83–5.65]	0.115		
60 and above	0.57 [0.09–0.43]	<0.001		

*Healthcare worker status*				
Healthcare professional	—			
Nonhealthcare worker	2.14 [1.56–2.93]	<0.001		

*Face mask use within last two weeks*				
Use any type of facemask	—			
Did not use face mask	0.17 [0.06–0.46]	0.001		

*Use of immune boosters*				
Did not take immune boosters	—			
Took immune boosters	2.65 [1.97–3.58]	<0.001		

AOR: adjusting for age, mask use, intake of immune booster, and healthcare worker status. Risk of infection and admission were associated with the method of administration of home-based remedies to prevent COVID-19.

**Table 5:** Logistic regression showing the odds of infection and admission associated with the method of administration of home-based remedies to prevent COVID-19.

Effect of mode of administration of home-based remedy on	Univariate logistic regression	Multivariate logistic regression	AOR [95% CI]	*p* value
	OR [95% CI]	*p* value		
Risk of infection of SARS-CoV-2 Steam inhalation Baths Drinks Food eaten or changes in diet Exercise	21.29 [5.03–90.2] 2.46 [0.58–10.4] 0.53 [0.34–0.84] 0.01 [0.00–0.08] 0.02 [0.00–0.18]	<0.001 0.220 0.007 <0.001 <0.001	26.63 [6.10–116.24] 2.69 [0.49–14.78] 0.67 [0.37–1.20] 0.01 [0.00–0.13] 0.02 [0.00–0.26]	<0.001 0.254 0.185 <0.001 0.003

*Risk of COVID-19-related admission* Drinks Food eaten or changes in diet	0.30 [0.09–0.97] 0.10 [0.01–0.70]	0.045 0.021	0.53 [0.13–2.09] 0.15 [0.02–1.17]	0.362 0.070

AOR: adjusting for age, mask use, immune booster use, and healthcare worker status. Gurgle had only two observations, and study not powered for subanalysis involving gurgle.

## Data Availability

The dataset used for this current study is available on reasonable request from the corresponding author.

## References

[1] Yuki K., Fujiogi M., Koutsogiannaki S. (2020). COVID-19 pathophysiology: a review. *Clinical Immunology*.

[2] WHO (2021). Director-General’s opening remarks at the media briefing on COVID. https://www.who.int/director-general/speeches/detail/who-director-general-s-opening-remarks-at-the-media-briefing-on-covid-19---11-march-2020.

[3] GHS Ghana (2021). COVID-19 updates. https://ghanahealthservice.org/covid19/.

[4] Nuertey B. D., Ekremet K., Haidallah A.-R. (2021). Performance of COVID-19 associated symptoms and temperature checking as a screening tool for SARS-CoV-2 infection. *PLoS One*.

[5] Weiss P., Murdoch D. R. (2020). Clinical course and mortality risk of severe COVID-19. *The lancet*.

[6] Yu X., Sun X., Cui P. (2020). Epidemiological and clinical characteristics of 333 confirmed cases with coronavirus disease 2019 in Shanghai, China. *Transboundary and emerging diseases*.

[7] Chu D. K., Akl E. A., Duda S. (2020). Physical distancing, face masks, and eye protection to prevent person-to-person transmission of SARS-CoV-2 and COVID-19: a systematic review and meta-analysis. *The lancet*.

[8] Di Matteo G., Spano M., Grosso M. (2020). Food and COVID-19: preventive/co-therapeutic strategies explored by current clinical trials and in silico studies. *Foods*.

[9] Remali J., Aizat W. M. (2021). A review on plant bioactive compounds and their modes of action against coronavirus infection. *Frontiers in Pharmacology*.

[10] Villena-Tejada M., Vera-Ferchau I., Cardona-Rivero A. (2021). Use of Medicinal Plants for COVID-19 Prevention and Respiratory Symptom Treatment during the Pandemic in Cusco, Peru: A Cross-Sectional Survey. *PLos One*.

[11] Belhaj S., Zidane L. (2021). Medicinal plants used to boost immunity and decrease the intensity of infection caused by SARS-COV-2 in Morocco. *Ethnobotany Research and Applications*.

[12] Ruiz-Padilla A. J., Alonso-Castro A. J., Preciado-Puga M. (2021). Use of allopathic and complementary medicine for preventing SARS-CoV-2 infection in Mexican adults: a national survey. *Saudi Pharmaceutical Journal*.

[13] World Health Organization (2021). *COVID-19 Clinical Management: Living Guidance, 25 January 2021*.

[14] Al Najrany S. M., Asiri Y., Sales I., AlRuthia Y. (2021). The commonly utilized natural products during the COVID-19 pandemic in Saudi Arabia: a cross-sectional online survey. *International Journal of Environmental Research and Public Health*.

[15] Chali B. U., Melaku T., Berhanu N. (2021). Traditional medicine practice in the context of COVID-19 pandemic: community claim in jimma zone, oromia, Ethiopia. *Infection and Drug Resistance*.

[16] Alzohairy M. A. (2016). Therapeutics role of Azadirachta indica (Neem) and their active constituents in diseases prevention and treatment. *Evidence-Based Complementary and Alternative Medicine*.

[17] Singh N. A., Kumar P., Jyoti N., Kumar N. (2021). Spices and herbs: potential antiviral preventives and immunity boosters during COVID-19. *Phytotherapy Research*.

[18] Ahkam A. H., Hermanto F. E., Alamsyah A., Aliyyah I. H., Fatchiyah F. (2020). Virtual prediction of antiviral potential of ginger (Zingiber officinale) bioactive compounds against spike and MPro of SARS-CoV2 protein. *Berkala Penelitian Hayati*.

[19] Safa O., Hassaniazad M., Farashahinejad M. (2020). Effects of Ginger on clinical manifestations and paraclinical features of patients with Severe Acute Respiratory Syndrome due to COVID-19: a structured summary of a study protocol for a randomized controlled trial. *Trials*.

[20] Tomani J. C. D., Kagisha V., Tchinda A. T. (2020). The inhibition of NLRP3 inflammasome and IL-6 production by hibiscus noldeae baker f. Derived constituents provides a link to its anti-Inflammatory therapeutic potentials. *Molecules*.

[21] Fernández‐Arroyo S., Herranz‐López M., Beltrán‐Debón R. (2012). Bioavailability study of a polyphenol-enriched extract from H ibiscus sabdariffa in rats and associated antioxidant status. *Molecular Nutrition & Food Research*.

[22] Zhen J., Villani T. S., Guo Y. (2016). Phytochemistry, antioxidant capacity, total phenolic content and anti-inflammatory activity of Hibiscus sabdariffa leaves. *Food Chemistry*.

[23] Al-Asmari A. K., Albalawi S. M., Athar M. T., Khan A. Q., Al-Shahrani H., Islam M. (2015). Moringa oleifera as an anti-cancer agent against breast and colorectal cancer cell lines. *PLoS One*.

[24] Flores-Félix J. D., Gonçalves A. C., Alves G., Silva L. R. (2021). Consumption of phenolic-rich food and dietary supplements as a key tool in SARS-CoV-19 infection. *Foods*.

[25] Wang F., Bao Y., Zhang C. (2021). Bioactive components and anti-diabetic properties of Moringa oleifera Lam. *Critical Reviews in Food Science and Nutrition*.

[26] Muhammad S., Hassan S. H., Al-Sehemi A. G. (2021). Exploring the new potential antiviral constituents of Moringa oliefera for SARS-COV-2 pathogenesis: an in silico molecular docking and dynamic studies. *Chemical Physics Letters*.

[27] da Silveira M. P., da Silva Fagundes K. K., Bizuti M. R., Starck É., Rossi R. C., de Resende e Silva D. T. (2021). Physical exercise as a tool to help the immune system against COVID-19: an integrative review of the current literature. *Clinical and Experimental Medicine*.

[28] Dixit S. (2020). Can moderate intensity aerobic exercise be an effective and valuable therapy in preventing and controlling the pandemic of COVID-19?. *Medical Hypotheses*.

[29] Heffernan K. S., Jae S. Y. (2020). Exercise as medicine for COVID-19: an ACE in the hole?. *Medical Hypotheses*.

[30] Aman F., Masood S. (2020). How Nutrition can help to fight against COVID-19 Pandemic. *Pakistan Journal of Medical Sciences*.

[31] Moscatelli F., Sessa F., Valenzano A. (2021). COVID-19: role of nutrition and supplementation. *Nutrients*.

[32] Uy T. M. Z., Miranda M. C. B., Aro S. J. V., Uy M. E. V. (2020). Should steam inhalation be used in the treatment and prevention of COVID-19. *Asia Pac Center Evid Based Healthcare*.

